# Student Feedback to Tailor the CARD™ System for Improving the Immunization Experience at School

**DOI:** 10.3390/children7090126

**Published:** 2020-09-04

**Authors:** Charlotte Logeman, Anna Taddio, C. Meghan McMurtry, Lucie Bucci, Noni MacDonald, Garth Chalmers, Victoria Gudzak, Vibhuti Shah, Joanne Coldham, Cheri Little, Tracy Samborn, Cindy Dribnenki, Joanne Snider

**Affiliations:** 1 Leslie Dan Faculty of Pharmacy, University of Toronto, Toronto, ON M5S 3M2, Canada; charlotte.logeman@sickkids.ca (C.L.); victoria.gudzak@mail.utoronto.ca (V.G.); 2 The Hospital for Sick Children, Toronto, ON M5G 1X8, Canada; 3 Department of Psychology, The University of Guelph, Guelph, ON N1G 2W1, Canada; cmcmurtr@uoguelph.ca; 4 McMaster Children’s Hospital, Hamilton, ON L8N 3Z5, Canada; 5 Immunize Canada, Canadian Public Health Association, Ottawa, ON K1Z 8R9, Canada; lbucci@cpha.ca; 6 Department of Pediatrics, Dalhousie University, Halifax, NS B3K 6R8, Canada; Noni.MacDonald@dal.ca; 7 University of Toronto Schools, Toronto, ON M5S 2R7, Canada; gchalmers@utschools.ca; 8 Department of Pediatrics, Mount Sinai Hospital, Toronto, ON M5G 1X5, Canada; Vibhuti.Shah@sinaihealth.ca; 9 Alberta Health Services, Calgary, AB T2W 3N2, Canada; Joanne.Coldham@albertahealthservices.ca (J.C.); Cheri.Little@albertahealthservices.ca (C.L.); Tracy.Samborn@albertahealthservices.ca (T.S.); Cindy.Dribnenki@albertahealthservices.ca (C.D.); Joanne.Snider@albertahealthservices.ca (J.S.)

**Keywords:** school immunizations, pain, fear, patient education, vaccine hesitancy, child, adolescent

## Abstract

Increasing the comfort of vaccine delivery at school is needed to improve the immunization experience for students. We created the CARD™ (C—Comfort, A—Ask, R—Relax and D—Distract) system to address this clinical care gap. Originally designed for grade 7 students, this study examined the perceptions of grade 9 students of CARD™. Grade 9 students who had experience with school-based immunizations, either as recipients or onlookers (*n* = 7; 100% females 14 years old) participated. Students answered pre–post surveys, reviewed CARD™ educational materials and participated in a semi-structured focus group discussion. The Consolidated Framework for Implementation Research (CFIR) was used as the framework for analysis of qualitative data. Participants reported positive perceptions of CARD™ educational materials and that CARD™ could fit into the school immunization process. CARD™ improved knowledge about effective coping interventions and was recommended for education of both nurses and students. The results provide preliminary evidence that CARD™ is acceptable and appropriate for implementation in grade 9 school-based immunizations.

## 1. Introduction

School-based (mass) immunizations are an efficient method of delivering vaccines to a large number of children. However, this approach is commonly associated with negative experiences in students related to associated fear, pain and fainting [1]. Negative experiences with immunization can lead to the development of needle fears [2,3,4]. In addition, they can contribute to immunization non-compliance and other health care-avoidance behaviours [4].

Overcoming barriers to immunization has been identified as a global priority by the World Health Organization [5]. While the reasons for refusing vaccination are multi-factorial, at present, concerns about pain and fear of needle injections are reported by one out of five adolescents that refuse immunizations [6]. Creating more positive experiences for students is therefore clinically relevant as it will promote vaccination. This is particularly important now, as the COVID-19 pandemic has prioritized research into the development of vaccines to prevent the spread of the disease. When a COVID-19 vaccine becomes available, it will be important to ensure that pain and fear are not barriers to its uptake.

In 2015, we published a Clinical Practice Guideline (CPG) that includes evidence-based recommendations for managing pain, fear and fainting during vaccine injections [7]. The CPG provides resources to guide implementation; however, they are not tailored to mass immunizations, such as immunizations at school. For many students, school immunizations are their first experience with having a painful procedure without parental support. At present, students are not routinely educated about what to expect or prepared for how to cope with this novel and painful event [8].

Adequate education and preparation of children for upcoming painful medical procedures is considered important [9]. Using the Knowledge-to-Action cycle [10] and the Consolidated Framework for Implementation Research (CFIR) [11] as the guiding frameworks, we created a Knowledge Translation (KT) tool to help translate the CPG recommendations for the school immunization setting. The KTA cycle describes translation of evidence into practice as the interplay between knowledge creation and action. CFIR provides a detailed list of constructs that influence implementation that can be used to develop and evaluate new innovations.

The KT tool, called the CARD™ (C—Comfort, A—Ask, R—Relax, and D—Distract) system, is a framework for delivering vaccinations at school that incorporates recommendations from the CPG. The word “CARD” itself is an easy-to-remember acronym for different categories of interventions students can choose from to help them to cope with pain, fear and fainting. For instance, interventions students can choose to ‘play’ from the Comfort and Distraction categories include bringing a support person, and using a cell phone, respectively [12]. Activities related to both the planning and delivery of vaccinations are altered to align with the principles of CARD™ and all individuals involved in the school immunization program are trained and versed in the framework, including students.

CARD™ contains educational resources targeted to students so that they are educated and prepared for upcoming school immunizations. In this study, we solicited the perspectives of grade 9 students about these educational materials. When we originally developed and evaluated the CARD™ educational materials, we included students in grades 6 to 8 [13,14,15,16]; however, across different geographical regions, immunizations are carried out in other grade levels, including grade 9 students. There are many transitions that students make in grade 9 that may impact on acceptability of CARD™. These include developmental as well as circumstantial changes, including increasing independence and interest in friends, changing schools, and increasing workload. The suitability of the original educational materials for grade 9 students needs to be established and/or adaptations are required before widespread implementation in older students. The specific objective was to explore grade 9 students’ perceptions of CARD™ for helping them cope with school immunizations. The results were used to inform broader implementation during grade 9 school immunizations in Calgary, Canada, in a cluster trial that is currently underway.

## 2. Materials and Methods

### 2.1. Design

This study included qualitative (focus group) and quantitative (survey) elements.

### 2.2. Participants and Setting

Seven female grade 9 students (age, 14 years) from a large independent school in Toronto participated in this study on 19 February 2019. The students had prior experience either as recipients (*n* = 6) or observers (*n* = 1) of school immunizations.

The parents and students of the entire grade 9 cohort (including male, female and non-binary) were invited to participate by a school administrator; the maximum number of participants allowed was 12. CARD™ had not been previously used at their school. This study was conducted in accordance with the declaration of Helsinki, and the protocol was approved by the research ethics board of the University of Toronto (#36893). Students were given a consent package to review with their guardians. All participants and their guardians signed the consent form before participation. This study was conducted at the school after school hours.

### 2.3. Materials and Procedures

We used similar procedures and instruments as in our prior studies [12,13,14,15,16,17]. First, students independently completed attitude surveys about immunization pain and fear and acceptance of immunizations. Then they completed a knowledge test about effective coping strategies during immunization. The knowledge test consisted of 10 dichotomous (yes/no) questions about the effectiveness of various strategies to reduce pain, fear and fainting during immunization. Students were asked to report whether they thought any of the ways described can help to make needles more comfortable, either by making the needle poke hurt less or by making it less scary. This could be for them or someone else. The number of correct answers was summed for a total score out of 10. Included questions were derived from the CPG [12]. The attitudes surveys used Likert scales to assess level of agreement with different statements about immunization pain and fear and acceptance of immunizations. In addition, students reported on their level of fear of immunization needles on a scale of 0 (no fear) to 10 (worst possible fear).

Students then participated in a focus group discussion that lasted approximately 1 h. The discussion was facilitated by a trained team member (AT) using a semi-structured interview guide. Students were asked about their experiences with school immunizations. Then they were shown key educational materials from the CARD™ system developed specifically for education and preparation of students, including 2 videos and 1 pamphlet. Of note, these materials were used during education of students in our prior work [12,13,14,15,16]. The first video (https://youtu.be/z57vTpb19wQ) reviews immunization—what it is, why it is needed, and what will happen. The second video (https://youtu.be/c41HvgEKQSk) reviews the CARD™ mnemonic (C—Comfort, A—Ask, R—Relax, and D—Distract) and includes vignettes of students undergoing immunization using the strategies based on the mnemonic. The pamphlet (included in reference Taddio et al. [12]) is a companion to the second (i.e., CARD™) video whereby students can record their preferred coping strategies for immunization. This pamphlet includes examples of coping strategies in each of the letter categories from which the students can choose. Students were asked to fill out the pamphlet with their own preferences for coping strategies for upcoming immunizations. Participants then provided feedback about these materials, including suggestions for changes. Students provided written feedback using a standardized survey that included statements about understandability and quantity of included information that they rated using Likert scales. After this, students repeated the attitude and knowledge surveys. Finally, participants completed a demographic survey that inquired about gender, age, and whether the student had experience with school-based immunizations.

### 2.4. Analytic Strategy

The focus group discussion was audiotaped and transcribed verbatim. The transcript was entered into NVivo (Version 10) for analysis and coded line-by-line using directed content analysis [18] using CFIR as the guiding framework. CFIR provides a menu of constructs that have been associated with effective implementation of complex interventions [11]. It provides a practical guide to assessing potential barriers and facilitators to CARD™ implementation based on theoretically-based constructs. For the purposes of coding, the inner and outer setting were collapsed into one domain called ‘setting’ in order to simplify the presentation. The transcripts were individually reviewed by three team members (CL, AT, VS). Coding was performed by all members and disagreements were resolved by consensus. In addition, triangulation of the different sources of data (qualitative and quantitative) was conducted. Together, these methods provided assurance for the credibility of the results. Quantitative data were analyzed using descriptive statistics (e.g., median, range). Pre- and post- attitudes survey responses, fear scores, and knowledge test scores were compared using the Wilcoxon Signed Rank test. This non-parametric statistic was selected because of the small sample size. The statistical program SPSS (version 24) was used to analyze the data. A *P*-value of <0.05 was considered significant.

## 3. Results

### 3.1. Qualitative Data

The data fit into two domains of CFIR: (1) intervention characteristics and (2) setting. They are described in the following section. Examples of quotes are provided in Table 1.

#### 3.1.1. Intervention Characteristics

##### Design Quality and Packaging (How the Intervention is Bundled, Presented and Assembled)

Students reported liking the educational resources. One student suggested making the videos shorter if they were going to be used at the time of immunization (rather than ahead of time). Students reported that they learned about immunization coping strategies from the CARD™ educational materials and about being able to use their preferred coping strategies.

##### Adaptability (Degree to Which an Intervention Can Be Adapted, Tailored, Refined or Reinvented to Meet Local Needs)

Students commented on the ability for the CARD™ system to be tailored to meet the needs of different students because of different preferences and that this should be stressed.

##### Relative Advantage (Perception of the Advantage of Implementing the Intervention versus an Alternative Solution)

Students identified ways that their immunization experience could be better with CARD™, including reducing anxiety and fear. Speaking openly about fear and normalizing fear were believed to be helpful.

#### 3.1.2. Setting

##### Culture (Norms, Values and Basic Assumptions)

Students reported that the nurses influenced their immunization experiences. Some students expressed that nurses did not use a student-centred approach when vaccinating students at school. They mentioned that nurses may require additional training regarding how to interact with students, including asking them about how they were feeling. Teachers were reported as having a secondary role in the immunization process.

##### Patient Needs and Resources (the Extent to Which Patient Needs, As Well As Barriers and Facilitators to Meet Those Needs, are Accurately Known and Prioritized by the Intervention Implementers)

Students mentioned that nurses were inconsistent in their approach when interacting with students: some were attentive to their needs and coached them while others did not. Some students mentioned that CARD™ would address some of the gaps in the immunization preparation process, such as education by nurses about how to cope. Students also reported that their preferred coping strategies were not previously accommodated, such as being immunized in private. Usual practice included being immunized in the gymnasium in front of peers; not all students were comfortable with this method.

##### Tension for Change (Degree to Which the Current Situation is Believed to Be Intolerable)

Some students reported that the current approach to immunizations should be changed. Students should be educated about vaccinations and ways to cope. Students should also be given reminders so that they dress in a manner that allows easy access to the upper arm for injections. Some students questioned why interventions were not offered proactively to make immunization a better experience.

##### Compatibility (How the Intervention Fits within Current Workflows and Systems)

Students reported that CARD™ interventions could fit into usual immunization interactions with nurses. For instance, nurses used verbal distraction and external distraction objects (e.g., stress balls).

### 3.2. Quantitative Data

Students’ attitudes and fear scores are displayed in Table 2. There were no significant changes from baseline to after review of the educational material (*p* > 0.05). Responses to the knowledge test are displayed in Table 3. Median total knowledge score after review of the educational materials was higher than at baseline: 8.5 (range, 5–10) vs. 6 (range, 4–8); *p* = 0.041. All seven students reported that they understood most or all of the information in both videos and the pamphlet and that the amount of information presented was just right.

## 4. Discussion

This small study examined the perspectives of grade 9 female students about the CARD™ system as a framework for delivering immunizations at school in order to inform future implementation of CARD™ during grade 9 mass immunizations. Specific educational materials directed to students were reviewed, including two videos and one companion pamphlet. Overall, the results demonstrated that students held positive attitudes about CARD™. Students learned about evidence-based coping strategies and reported that CARD™ filled an important clinical care gap related to addressing pain and fear during immunization. They encouraged education of students as well as adults involved in the immunization delivery process, particularly nurses, to ensure that they are versed in CARD™ and can adequately support students. Students recommended the integration of CARD™ into usual practices.

These results are consistent with prior studies conducted by our team with children [13,14,15,16]. In these studies, grade 6–8 student participants reported that all students should be educated about CARD™ as well as adults. This would help create a more supportive and positive environment during immunization. Further, prior studies across these grade levels demonstrated an increase in student knowledge about effective coping interventions after reviewing CARD™ educational material. Finally, in a controlled clinical trial including grade 7 students undergoing school-based immunizations, CARD™ reduced fear and dizziness (precursor of fainting) during vaccine injections [16]. It is important to note that in work leading up to the creation of CARD™ carried out by our team, children stressed the importance of being prepared ahead of time and being able to choose coping interventions [19]. In creating CARD™, students were involved in all phases, informing the content and delivery method for the included information [13,14,15,16]. This likely contributed to the positive effects observed with its use when implemented during grade 7 immunizations [16].

Students in the present study highlighted the influence of a nurse’s qualities on their experience with immunization. Separately, in another study of the factors affecting the decision to seek health care, qualities of a health care professional were deemed important for engagement of adolescents, including honesty, respectfulness, empathy, competency, and fair treatment [20]. Through the implementation of CARD™, nurses build on these qualities and align their activities with currently espoused models of care delivery such as person-centred care [21]. In fact, feedback obtained from children across studies on this topic are consistent with a person-centred care approach.

There are some limitations worthy of discussion. The primary one relates to the small sample size, which not only limited our ability to detect statistically significant differences in some quantitative outcomes, but also limited our ability to ensure that a wide range of opinions about CARD™ were included. Sampling was limited to one independent school and participants included female students only (i.e., no male students agreed to participate). It is possible that students in other schools (and regions) as well as male students may have different perceptions. Given that female students report more pain and fear, this may have led to more positive attitudes overall [22]. Second, the focus group may have been biased by social interaction. Outspoken group members may have heavily influenced others. Third, the way in which CARD™ was introduced and taught to the students in this study differs from how it is intended to be implemented in the real world—focus group interview vs. large classroom lesson. Our study is unable to determine perceptions of students to different approaches to education delivery and other aspects of program delivery, including the timing of education relative to the day of immunization and the influence of peers, teachers, public health nurses or parents [23,24,25,26]. Studies are currently underway to examine the influence of such factors on implementation outcomes.

There are several strengths of this study. Firstly, we involved the primary clients for school-based immunizations—students—in helping to inform implementation of CARD™. This approach is aligned with user-centred approaches to research [27]. Despite the limited number of participants, individuals were included that reported varying levels of fears (ranging from 0 to 9 on a 0 to 10 scale), and both positive and negative experiences with school-based immunizations. This suggests that individuals with a diversity of perspectives on the topic were included. In addition, we involved multiple coders in the analysis, and found consistent results across qualitative and quantitative data sources, improving the credibility of the results [28].

In summary, this study provides the first evidence of the acceptability of CARD™ in grade 9 students. The results confirm that CARD™ is acceptable to grade 9 students and no specific changes were made as a result of this study. Together with the results of a subsequent study documenting the feasibility of CARD™ [29], a large cluster trial has begun examining the effectiveness and fidelity of implementation in schools in Calgary, Canada. CARD™ systematically incorporates evidence-based interventions in school immunization program planning and delivery activities that include educating and preparing students about immunizations and coping strategies ahead of time. Students are actively engaged and learn skills they can use to manage how they feel during immunization. Educating and preparing students can lead to more positive experiences and attitudes about immunization and greater engagement in health care decision making in the future. Individuals involved in school immunizations, including parents, teachers, and nurses, can use the results from this study to reflect on their own practices related to immunizations and determine whether changes are warranted. Importantly, frameworks for vaccine delivery that are person centred such as CARD™ have the potential to promote immunization uptake across immunization settings. Given that the threat of life-threatening infectious diseases is always present, as evidenced by the current pandemic, this work is particularly relevant as, when a COVID-19 vaccine becomes available, efforts need to be made to ensure that pain and fear are not barriers to its acceptance.

## Figures and Tables

**Table 1:** Selected quotes from focus group discussion with students after review of educational materials (*n* = 7).

Domain	Sample Quote
Intervention characteristics	
Design Quality and Packaging	I feel like these videos are super useful. And I feel like, the concepts of these should be more widely broadcast because the whole thing about vaccines and why people get so anxious is a big part of it… you feel stupid for getting scared, right? (Student 2)
	I think that the videos are really good in the sense that it would make me more comfortable if I was listening to that before I would get a vaccination … If I had any inquiries or like, if I was nervous about it. It shows footage of kids getting the vaccinations then you see, oh maybe it won’t be that bad. But the only thing I suggest is that the videos were kind of long. So, I don’t know if I have the patience. Like, if I was like anxious about getting a vaccine right before, to listen to all of that. So, they should make it more concise. (Student 3)
	“I never knew that was an option or even available and I didn’t think the nurse giving me the needle would allow me to listen to music because no one’s ever brought that up to me.” (Student 6)
Adaptability	I feel like in the videos, it did like, iterate this… for everyone it’s going to be different. So, I feel like they really have to stress that point. (Student 2)
Relative Advantage	Next time I might be like less anxious because, like, I have these methods I can use that I have not tried before. (Student 4)
	I feel like integrating this type of, like, language that normalises coping techniques, instead of like just being like - Are you scared?- would definitely help with people; not only feeling okay with being scared … so like they’re not trying to cover up anything but also in… just in general, I feel like when you know that people aren’t judging you for being scared, you get like, more comfortable. (Student 2)
Setting	
Culture	It just kind of seems like, whenever I’ve gotten them administered to me, they just kind of want to finish. It’s not about making it better for you, it’s about making it better for them and more convenient for them to get over their job. And I don’t think that’s right. It’s almost like whenever I ask a question towards, specifically vaccines, they seem annoyed. Like, they shouldn’t be annoyed if I’m going to be asking you why I’m going to get this or what it’s going to do to me. (Student 3)
	I feel like its one thing to be informed about the information but also another side, to also educate the nurse on what they’re supposed to say. Like there should be a designated script: *How do you feel?* Standard questions that they should be asking each and every person that they vaccinated, right? So, I think its not just a part of us to be aware of what’s happening. Its a part of them to just know what is going to happen … what to do. Because its their responsibility. (Student 3)
	Yea, the teachers were, I think, just mostly supervising but if anyone like, did need a little bit of help, they did come in and ask them if they were okay. (Student 3)
Patient Needs and Resources	One time, I was like, given the vaccine by someone who was like, very nice and gave me a stress ball and was like: *Don’t worry, just focus on doing that and just like, it’ll be over soon…* He was very nice and very attentive to how I was feeling. I remember the following year someone was giving me the vaccine and I was so nervous. I kind of used a little humour to kind of, laugh it off, and they didn’t really seem to care if I was like, anxious or not… I was obviously pretty anxious. I just think its really important. Like my experience was much better when it was someone who was obviously engaged in trying to help you. (Student 4)
	I really agree with (another participant) that like, the person injecting or giving the vaccine, they have a responsibility to not only like, give the vaccine, but…give them methods to cope with any fear or like, how to make it hurt less or whatever… it’s very important to be able to work with the person and let them know what’s happening. (Student 4)
	I kind of get a little nervous if I’m in front of a really large group of people. I do appreciate kind of having emotional support with my friends but like the fact that I’m with a lot of people does make me kind of nervous. And I don’t really like, like that kind of environment. (Student 6)
Tension for Change	Many people are like afraid of needles and I think this should be addressed, or like a fear of just what’s happening to their bodies... They should be well informed about these kinds of things. (Student 4)
	I didn’t know I was going to get a vaccination. Um, luckily, I’m not someone who gets nervous about vaccinations or needles and things like that. So, I was fine. But I could imagine other people in my situation wouldn’t like it that much. I was also really lucky because the person giving me the vaccination was very clear about exactly what was happening. They gave me a count down. They told me exactly what they were giving me, which in my experience, usually doesn’t happen. And I found a lot of my friends who did get vaccinations weren’t aware of what was happening and what kind of vaccinations they were getting. Um, and I know like from experience, I’m kind of a shy person, so, I don’t know if I would even have the courage to ask them if they hadn’t like, made it clear to me. And so, I think that’s really important to just let students know, like beforehand, that they’re going to get a vaccination. To prepare for that. Because I also know that there was a situation, for a lot of girls, um, their sleeves weren’t rolled up - wouldn’t roll up the whole way. So, its kind of uncomfortable and they had to go to a different room and get some privacy, so they could take their whole shirt off. And I could only imagine how uncomfortable that would be. So I think that’s also really important. (Student 6)
	There was this girl who was like, really anxious about getting a vaccination to the point that I’m pretty sure she had a panic attack [on the day]. And it was only at that point that they offered the numbing patch to her, which she did get. She could not have it. Like, she was freaking out. Um, and so they were like: *Okay, well like, what are you scared about?* And she was like: *I’m just really scared about the pain. I’m freaking out.* And they were like: *Okay we have a numbing patch - do you want that?* And so, they did that, but like only after she had been having like, like having really severe anxiety. So, I feel like it should be introduced as an option like, perhaps on the form - so people know at least - because I hadn’t known about it until she told me about it. (Student 2)
Compatibility	They were making sure that like, everything was okay. Like, I didn’t really, I don’t think I showed any signs of being like, really anxious or stressed. They were just checking in and, um, said: *If you’re dizzy, let us know, like if you feel off, just come up to one of us and we’ll take care of it.* (Student 6)
	I was very anxious. When I went there, the man giving me the needle, he gave me a stress ball to hold with like, one hand, and he put the needle in the other one. (Student 4)

**Table 2:** Student attitudes about immunization and pain and fear before and after review of educational materials (*n* = 7) *.

	Score at Baseline Median (Range)	Score after Review Median (Range)
**Attitudes about pain and fear** **Scores range from 1 to 5 (1 = strongly disagree, 2 = disagree, 3 = no opinion, 4 = agree, and 5 = strongly agree)**		
Vaccine injections cause the same amount of pain in everybody	2.0 (2.0–4.0)	1.0 (1.0–2.0)
I am satisfied with how my pain has been managed during vaccine injections	4.0 (3.0–5.0)	3.0 (2.0–4.0)
We don’t need to do anything about students’ pain during vaccine injections at school because pain is a normal part of the procedure	2.0 (3.0–5.0)	2.0 (3.0–5.0)
Doctors and nurses should help make vaccine injections at school less painful for children	4.0 (3.0–5.0)	5.0 (4.0–5.0)
Parents and students should be given information about how to make vaccine injections at school less painful for students	4.0 (3.0–5.0)	5.0 (4.0–5.0)
Teachers should be given information about how to make vaccine injections at school less painful for students	4.0 (3.0–5.0)	5.0 (3.0–5.0)
Vaccine injections cause the same amount of fear in everybody	1.0 (1.0–2.0)	1.0 (1.0–2.0)
I am satisfied with how my fear has been managed during vaccine injections	4.0 (2.0–4.0)	3.0 (2.0–4.0)
We don’t need to do anything about students’ fear during vaccine injections at school because fear is a normal part of the procedure	1.0 (1.0–3.0)	2.0 (1.0–2.0)
Doctors and nurses should help make vaccine injections at school less scary for students	5.0 (4.0–5.0)	5.0 (4.0–5.0)
Parents and students should be given information about how to make vaccine injections at school less frightening for students	5.0 (4.0–5.0)	5.0 (4.0–5.0)
Teachers should be given information about how to make vaccine injections at school less frightening for students	4.0 (3.0–5.0)	5.0 (4.0–5.0)
**Fear of immunization needles** Scores range from 0 to 10 (where the anchors are: 0 = no fear and 10 = worst possible fear)		
How afraid are you of getting immunization needles?	5.0 (0.0–9.0)	5.0 (0.0–7.0)
**Attitudes about immunization** Scores range from 0 to 4 (0 = no, 1 = maybe no, 2 = I don’t know, 3 = maybe yes, and 4 = yes)		
Do you think you should get immunized?	4.0 (4.0)	4.0 (4.0)
Scores range from 0 to 3 (0 = of course they should not, 1 = maybe they should not, 2 = maybe they should, and 3 = of course they should)		
Do you think family or friends should get immunizations?	3.0 (3.0)	3.0 (3.0)

* Wilcoxon Signed Rank Test: *p* > 0.05 for all analyses.

**Table 3:** Student knowledge scores before and after review of educational materials (*n* = 6) *^†^.

Strategies That Can Help to Make Needles More Comfortable, Either by Making the Needle Poke Hurt Less or by Making It Less Scary. The Correct Response Is ‘Yes’ for All Statements.	Frequency of Correct Answers at Baseline (%)	Frequency of Correct Answers after Review (%)
Have someone with you like a parent or friend	5 (83)	5 (83)
Have privacy so people cannot see each other getting the needle	3 (50)	6 (100)
Use medication to numb the skin so you don’t feel the needle	5 (83)	5 (83)
Distract yourself so you are paying attention to something else	4 (67)	6 (100)
Relax by taking deep belly breaths to help you stay calm	3 (50)	3 (50)
Ask questions so you know what will happen	3 (50)	6 (100)
Relax the arm getting the needle so that it is jiggly like spaghetti	3 (50)	4 (67)
Sit down in a comfortable position	6 (100)	6 (100)
Look away from the needle	3 (50)	4 (67)
Make your legs and tummy muscles tight (or tense) so you don’t feel dizzy	2 (33)	3 (50)

* N = 1 missing; **^†^** Wilcoxon Signed Rank Test: median total knowledge score after review of educational materials was higher than at baseline: 8.5 (range, 5–10) vs. 6 (range, 4–8); *p* = 0.041.
